# Pulse oximeter measurement error of oxygen saturation in patients with SARS-CoV-2 infection stratified by smoking status

**DOI:** 10.1183/13993003.01190-2022

**Published:** 2022-11-17

**Authors:** Colin J. Crooks, Joe West, Joanne R. Morling, Mark Simmonds, Irene Juurlink, Steve Briggs, Simon Cruickshank, Susan Hammond-Pears, Dominick Shaw, Timothy R. Card, Andrew W. Fogarty

**Affiliations:** 1 Nottingham Digestive Diseases Centre, School of Medicine, University of Nottingham, Nottingham, UK; 2 NIHR Nottingham Biomedical Research Centre (BRC), Nottingham University Hospitals NHS Trust and the University of Nottingham, Nottingham, UK; 3 Nottingham University Hospitals NHS Trust, Nottingham, UK; 4 Lifespan and Population Health, School of Medicine, University of Nottingham, Nottingham, UK; 5 East Midlands Academic Health Science Network, University of Nottingham, Nottingham, UK; 6 NIHR Biomedical Respiratory Research Centre University of Nottingham, Nottingham, UK

## Abstract

As pulse oximeters are now so widely used, it is important to identify any patient groups in whom they may also have a systematic bias that may impair the delivery of medical care to these individuals. One group would be tobacco smokers [1–3], as the inhaled carbon monoxide modifies the haemoglobin molecule within 1–2 min of inhaling tobacco smoke [4], and the subsequent increase in blood carboxyhaemoglobin levels modifies the pulse oximetry signal [5]. This was reported in a series of 16 patients with carbon monoxide poisoning from 1994 which resulted in higher pulse oximetry measurements than the true values, with the comment that this phenomenon may also extend to oxygen saturation measured in smokers as well [6]. To date, no robust real-world clinical data on acutely unwell patients exist to clarify the impact of smoking status and blood carboxyhaemoglobin levels on the measurement error of oxygen saturation by pulse oximeters.

## Abstract 2

**There is substantial measurement error in pulse oximetry readings of oxygen saturation below 90% which is not due to smoking status** https://bit.ly/3RunKtL

*To the Editor*:

Pulse oximeters provide a non-invasive measurement of oxygen saturation and are now routinely used to assess patients, inform medical decision-making and monitor subsequent clinical status.

As pulse oximeters are now so widely used, it is important to identify any patient groups in whom they may also have a systematic bias that may impair the delivery of medical care to these individuals. One group would be tobacco smokers [1–3], as the inhaled carbon monoxide modifies the haemoglobin molecule within 1–2 min of inhaling tobacco smoke [4], and the subsequent increase in blood carboxyhaemoglobin levels modifies the pulse oximetry signal [5]. This was reported in a series of 16 patients with carbon monoxide poisoning from 1994 which resulted in higher pulse oximetry measurements than the true values, with the comment that this phenomenon may also extend to oxygen saturation measured in smokers as well [6]. To date, no robust real-world clinical data on acutely unwell patients exist to clarify the impact of smoking status and blood carboxyhaemoglobin levels on the measurement error of oxygen saturation by pulse oximeters.

We have explored the association between smoking status and measurement error for pulse oximetry measurements of oxygen saturation.

The study used routinely collected electronic data for patients admitted to Nottingham University Hospitals NHS Trust between 1 February 2020 and 31 December 2021 with either a clinical or a microbiological diagnosis of severe acute respiratory syndrome coronavirus 2 (SARS-CoV-2) infection.

Oxygen saturations are recorded electronically. Pulse oximetry measurements with a paired blood gas measurement of oxygen saturations within a 30-min time window were used as the primary outcome. Oximetry measurements taken in intensive care units were not included in this study. All patients who are admitted to this institution are asked their smoking status at admission, and these data are also recorded electronically.

A mixed effects linear model was used for the primary analysis of the association between smoking and measurement error of oxygen saturation obtained by pulse oximetry methodology adjusting for age, sex and ethnic group [7]. Carboxyhaemoglobin is an objective measure of recent air pollution exposure [4], and this was also modelled separately.

Approval for this work was granted *via* an NUH Clinical Effectiveness Team audit (reference: 21-294C) and IRAS (REC: 20/WM/0142, project ID: 282490, amendment number SA02 20/07/21).

Data were available from 3266 patients with 5503 paired oxygen saturations (excluding arterial or pulse oximetry oxygen saturation measurements ≤70%), from both pulse oximetry and an arterial blood gas within a 30-min interval (4285 non-smokers *versus* 1218 smokers). The pulse oximetry arterial oxygen saturation difference (pulse oximetry minus arterial blood gas oxygen saturation measurements) for paired measurements was calculated and this was the outcome measure of interest.

Smokers were younger than non-smokers (median age 62 years (interquartile range (IQR) 51–72 years) *versus* 72 years (IQR 59–81 years); p<0.0001, Kruskal–Wallis test) and were less likely to be male (49% *versus* 57%; p<0.0001, chi-square test). Smokers were also more likely to be of white ethnicity (smokers 81% white *versus* non-smokers 72% white; p<0.0001, chi-square test) and had lower body mass indices (smokers median 25 kg·m^−2^ (IQR 21–32 kg·m^−2^) *versus* non-smokers 28 kg·m^−2^ (IQR 24–33 kg·m^−2^); p<0.0001, Kruskal–Wallis test). Median oxygen saturation from pulse oximetry was lower in smokers compared to non-smokers (93% (IQR 90–96%) *versus* 94% (IQR 91–96%); p<0.0001, Kruskal–Wallis test), as was arterial blood gas (93.1% (IQR 88.9–96.2%) *versus* 94.0% (IQR 90.5–96.8%); p<0.0001, Kruskal–Wallis test). Median arterial carboxyhaemoglobin was higher in smokers than non-smokers (1.6% (IQR 1.1–2.1%) *versus* 1.1% (IQR 0.8–1.5%), respectively; p<0.0001, Kruskal–Wallis test).

The initial regression model to assess the association between current smoking and the difference between pulse oximetry and arterial blood gas measures of oxygen saturation was adjusted for age, sex and ethnicity. In smokers, pulse oximetry resulted in an overestimation of arterial oxygen saturation by +0.7% (95% CI +0.3% to +1.1%), while a unit increase in carboxyhaemoglobin levels was also associated with an overestimation of oxygen saturation by pulse oximeters (+0.7%, 95% CI +0.5% to +0.9%). However, adjustment for oxygen saturation as measured by arterial blood gases eliminated both of these associations, giving values of −0.05% (95% CI −0.4% to +0.3%) and +0.04% (95% CI −0.1% to −0.2%) for smoking and carboxyhaemoglobin respectively. A sensitivity analysis of available oxygen saturation data gave similar qualitative associations. There was minimal evidence to support including an interaction between the measurement error with smoking and the arterial oxygen saturation level (p=0.5).

This suggests the apparent increased measurement error of oxygen saturation readings from pulse oximeter in smokers that was observed in the initial model is due to confounding by their lower oxygen saturation levels, where the measurement error of oxygen saturation is higher. This phenomenon can be seen in figure 1, where there is larger measurement error in the lower oxygen saturation categories, which are more likely to consist of smokers compared to the higher oxygen categories (chi-square test, p<0.0001).

**FIGURE 1:**
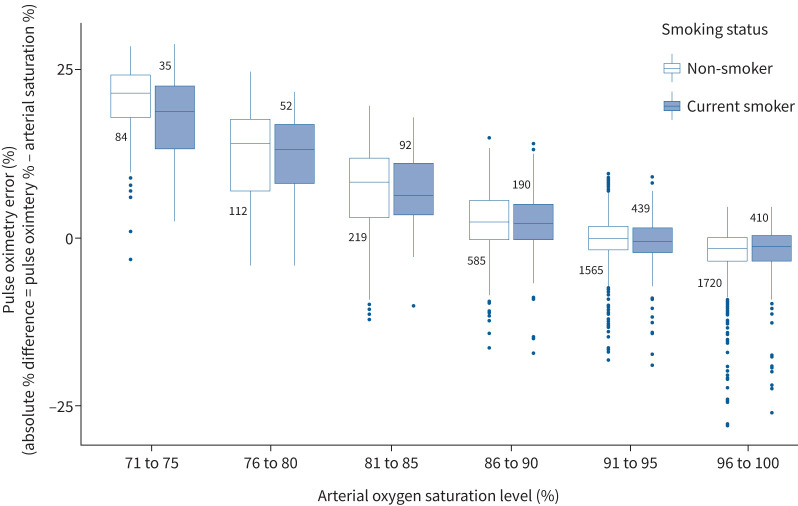
Measurement error of oxygen saturation as measured by pulse oximetry across a range of oxygen saturations measured by arterial blood gases stratified by smoking status. Boxes represent interquartile ranges (IQR), midpoint lines are median values, whiskers are IQR+1.5×IQR, and dots are outliers beyond the whiskers.

The strengths of our data are the large study population, which included all admissions over a defined time period with a diagnosis of SARS-CoV-2 infection, allowing us to adjust for potential confounding factors including a wide range of oxygen saturations. The ability also to use data on carboxyhaemoglobin levels allowed us to use an objective marker of air pollution, which is considered to be on the causal pathway of any potential association between smoking [5] and measurement error of oxygen saturation as measured by pulse oximetry. The real-world nature of these data means that the 30-min interval between the two measurements of oxygen saturation will introduce some measurement error into the analysis, we are unable to identify the distinct oximeters that generated the readings used in the analysis, or have objective assessment on whether there was ongoing use of tobacco during the hospital stay (which would be contrary to hospital regulations).

The clinical implications of these data are that there is no observable association between current smoking status and measurement error that is directly attributable to smoking tobacco. However, as a consequence of having more advanced lung disease, smokers have lower oxygen saturation levels which itself results in more measurement error of oxygen saturation from pulse oximetry for this category of patients. The prevalence of smoking for UK emergency respiratory admissions is 30% [8], and hence this indirect effect will impact on large numbers of patients as even a small overestimate in oxygen saturation readings may delay timely initiation of treatment in a large number of patients. As can be seen in figure 1 the potential size of this measurement error is large once oxygen saturation decreases below 85%, and hence this suggests that direct measurement using an arterial blood gas sample should be considered. These data are also relevant for researchers who wish to study factors that are associated with lung disease on pulse oximetry measurement error, as not adjusting for oxygen saturation levels may give erroneous results.

## Shareable PDF

10.1183/13993003.01190-2022.Shareable1 This one-page PDF can be shared freely online.
Shareable PDF ERJ-01190-2022.Shareable
