# Supplementary material for: Pulse oximeter measurement error of oxygen saturation in patients with SARS-CoV-2 infection stratified by smoking status
Source: Eur Respir J. 2022 Nov 17;60(5):2201190. doi: 10.1183/13993003.01190-2022 (PMC9558426; doi:10.1183/13993003.01190-2022)
Supplement: Supplementary file 1 [file ERJ-01190-2022.Shareable.pdf]

EUROPEAN RESPIRATORY JOURNAL
RESEARCH LETTER

SHAREABLE PDF

Pulse oximeter measurement error of oxygen saturation in patients with SARS-CoV-2 infection stratified by smoking status

Colin J. Crooks[1,2,3], Joe West[2,3,4,5], Joanne R. Morling[2,3,4], Mark Simmonds[3], Irene Juurlink[3], Steve Briggs[3], Simon Cruickshank[3], Susan Hammond-Pears[3,5], Dominick Shaw[3,6], Timothy R. Card[2,3,4] and Andrew W. Fogarty[2,3,4]

[1]Nottingham Digestive Diseases Centre, School of Medicine, University of Nottingham, Nottingham, UK. [2]NIHR Nottingham Biomedical Research Centre (BRC), Nottingham University Hospitals NHS Trust and the University of Nottingham, Nottingham, UK. [3]Nottingham University Hospitals NHS Trust, Nottingham, UK. [4]Lifespan and Population Health, School of Medicine, University of Nottingham, Nottingham, UK. [5]East Midlands Academic Health Science Network, University of Nottingham, Nottingham, UK. [6]NIHR Biomedical Respiratory Research Centre University of Nottingham, Nottingham, UK.

Corresponding author: Andrew W. Fogarty (andrew.fogarty@nottingham.ac.uk)

Check for updates

Shareable abstract (@ERSpublications)
There is substantial measurement error in pulse oximetry readings of oxygen saturation below 90% which is not due to smoking status https://bit.ly/3RunKtL

Cite this article as: Crooks CJ, West J, Morling JR, *et al*. Pulse oximeter measurement error of oxygen saturation in patients with SARS-CoV-2 infection stratified by smoking status. *Eur Respir J* 2022; 60: 2201190 [DOI: 10.1183/13993003.01190-2022].

This single-page version can be shared freely online.

Copyright ©The authors 2022.

This version is distributed under the terms of the Creative Commons Attribution Licence 4.0.

Received: 26 April 2022
Accepted: 26 Sept 2022

To the Editor:

Pulse oximeters provide a non-invasive measurement of oxygen saturation and are now routinely used to assess patients, inform medical decision-making and monitor subsequent clinical status.
